# Diagnosis and Study of Mechanical Vibrations in Cargo Vehicles Using ISO 2631-1:1997

**DOI:** 10.3390/s23249677

**Published:** 2023-12-07

**Authors:** Alejandro Medina Santiago, Jorge Antonio Orozco Torres, Carlos Arturo Hernández Gracidas, Salvador Hernández Garduza, Javier Duarte Franco

**Affiliations:** 1 CONAHCYT-Instituto Nacional de Astrofísica, Óptica y Electrónica, Coordinación de Ciencias Computacionales, San Andrés Cholula 72840, Puebla, Mexico; 2 TecNM/Instituto Tecnológico de Tuxtla Gutiérrez, Departamento de Ingeniería Industrial, Departamento de Desarrollo Académico, Tuxtla Gutiérrez 29050, Mexico; jorge.ot@tuxtla.tecnm.mx (J.A.O.T.); salvador.hg@tuxtla.tecnm.mx (S.H.G.); 3 Facultad de Ciencias Físico Matemáticas, CONAHCYT-Benemérita Universidad Autónoma de Puebla, Puebla 72570, Mexico; cahernandezgr@conahcyt.mx; 4 Instituto Politécnico Nacional, Escuela Superior de Ingeniería Mecánica y Eléctrica Unidad Zacatenco (ESIME), Ingeniería en Sistemas Automotrices, Ciudad de México 07738, Mexico; duartef23javier@gmail.com

**Keywords:** ISO 2631-1:1997, mechanical vibration, electronic, graphical interface, vibration diagnosis

## Abstract

This study presents the design and implementation of an electronic system aimed at capturing vibrations produced during truck operation. The system employs a graphical interface to display vibration levels, ensuring the necessary comfort and offering indicators as a solution to mitigate the damage caused by these vibrations. Additionally, the system alerts the driver when a mechanical vibration that could potentially impact their health is detected. The field of health is rigorously regulated by various international standards and guidelines. The case of mechanical vibrations, particularly those transmitted to the entire body of a seated individual, is no exception. Internationally, ISO 2631-1:1997/Amd 1:2010 oversees this study. The system was designed and implemented using a blend of hardware and software. The hardware components comprise a vibration sensor, a data acquisition card, and a graphical user interface (GUI). The software components consist of a data acquisition and processing library, along with a GUI development framework. The system underwent testing in a controlled environment and demonstrated stability and robustness. The GUI proved to be intuitive and could be integrated into modern vehicles with built-in displays. The findings of this study suggest that the proposed system is a viable and effective method for capturing vibrations in trucks and informing drivers about vibration levels. This system has the potential to enhance the comfort and safety of truck drivers.

## 1. Introduction

Various international standards and guidelines meticulously govern the health-related field. The case of mechanical vibrations, specifically those that are transmitted to the whole body of a seated person, is no exception. The drivers of tractor-trailers that circulate daily on the roads of Mexico are some of those who are exposed to these types of mechanical vibrations. An alternative to prevent health effects is to warn, in advance, through a graphical interface, that the mechanical vibration is exceeding the limits established in ISO 2631-1:1997 [1].

A mechanical vibration is the back-and-forth or up-and-down movement of an object near a position. For example, the beating of a heart, the screeching of a cicada, the swinging of a pendulum, or the small vibrations of mechanical parts of a machine in operation are all examples of mechanical vibrations [2]. Mechanical vibration, a key factor affecting comfort, must be considered from the design stage. Driving comfort in motor vehicles is affected from the floor to the driver’s seat [3].

The negative consequences for drivers after long working days of driving trucks on the roads are significant health deterioration, developing musculoskeletal disorders, and degenerative changes in the lumbar spine [4,5]. The mechanical vibration that can be transmitted to the whole body by a surface (seat) during the operation of a cargo vehicle (tractor-trailer) over time can have very serious health consequences. These consequences depend on two factors: acceleration and frequency.

The manufacturers of heavy vehicles take care of this issue from the moment the vehicle is designed. However, after the vehicle has traveled a certain number of kilometers on the very rough roads of Mexico, it is no longer guaranteed that the mechanical vibration transmitted to the driver is below the established limit. The main anti-vibration systems are the seats, which partly isolate the driver from mechanical vibration between the passenger compartment and the seat. These seats have a limited service life and can deteriorate rapidly. At present, there is no system on board a cargo vehicle that determines when there is a harmful vibration for the driver. This is why we propose this work, with the development in the laboratory of a robust prototype to obtain measurements.

Controversial and divergent hypotheses:

- Effects of mechanical vibrations on drivers: the claim that mechanical vibrations, especially those experienced by truck drivers, have significant negative health consequences, including musculoskeletal disorders and degenerative changes in the lumbar spine, could be debated, as different studies and perspectives may question the severity of these effects.
- Effectiveness of anti-vibration systems: the long-term effectiveness and durability of anti-vibration systems, in this case, seats, as a means of mitigating the adverse effects of mechanical vibrations, can be a point of controversy. It remains uncertain whether such systems truly deliver anticipated results over time and with continued use.

The primary objective of this study is to explore and evaluate these issues by creating a durable prototype that can obtain reliable real-time measurements of mechanical vibrations and provide a diagnostic system to identify when these vibrations exceed the limits specified by ISO 2631-1:1997 [1].

Main conclusions:

- Limitations of current anti-vibration systems: current anti-vibration systems, especially seats, demonstrate limited durability and may lose effectiveness over time, which could affect the health of drivers.
- Need for an onboard diagnostic system: the absence of an onboard system in a cargo vehicle, which identifies the occurrence of harmful vibrations to the driver, emphasizes the requirement for an innovative approach, such as the one developed in this study, that offers precise measurements and timely alerts.
- Importance of considering the design phase: this research highlights the importance of considering mechanical vibrations from the design phase of vehicles, suggesting the possibility of implementing design changes to minimize adverse health effects on drivers.

This paper seeks to examine the controversies surrounding the impact of mechanical vibrations on driver health objectively. It questions the effectiveness of current anti-vibration systems, while proposing a new diagnostic and preventative approach to tackle health problems associated with said vibrations.

The organization of this paper is as follows. Section 2 presents the state of the art of our article. Section 3 defines the design methodology in its structure and architecture of the data acquisition system. Section 4 shows the numerical results of the diagnostic system, based on the programming pseudocode. The discussion of the results is presented in Section 5. Finally, Section 6 presents the conclusions.

## 2. Background

Mechanical vibrations that can be transmitted to the whole body by a surface (seat) during the operation of a cargo vehicle (e.g., a tractor-trailer) can have serious health consequences over time. One way to prevent these consequences is to use an onboard system with a graphical interface that alerts the driver when there is a mechanical vibration that could have a negative impact on their health. This vibration is evaluated based on equation 1 of ISO 2631-1:1997, considering an average working day of 8 h [1].

The graphical interface, operating in real time, will inform the driver about the state of the air spring. Some authors have suggested that air springs have two positions in which mechanical vibration is not attenuated: the lower and upper positions. Therefore, the graphical interface will give two warnings to the driver and suggest avoiding these two states of the seat.

The authors of [6] mention that the design of each seat must be studied millimeter by millimeter to offer a perfect trade-off between firmness and comfort. The exact density of the foam used in the edge of the seat is carefully studied to ensure it supports an average person’s weight, ensuring it is neither too hard and uncomfortable nor too soft, causing the person to sink in.

Since the inception of cushioned seats, significant time has passed, and research continues to focus daily on enhancing the in-vehicle experience for occupants [7]. The seat should be designed with good lumbar support, balanced padding, and cushioning to absorb the jolts that occur on uneven pavements. Inadequate seating will lead to increased fatigue and back pain.

Conversely, contemporary advancements involve integrating electronic and electrical systems into the seats, enabling electrical readjustments that can be either manual or automatic. The less advanced systems usually have few readjustments (in horizontal displacement, height, and tilt), but the most modern and complete ones can have up to 16 different electrical readjustments, allowing the height, tilt, and displacement of the seat, as well as the inclination of the backrest, to be individually regulated [8].

### Studies on Mechanical Vibrations

Currently, studies are being conducted on various types of vehicles to ascertain if, in line with existing ISO standards on whole-body exposure to mechanical vibrations, there could be potential harm to a worker’s health. Some of these studies are shown below.

In [9], the author discussed a study of the exposure to mechanical vibrations in the different workplaces of mining companies affiliated with the ACHS (Chilean Safety Association). The equipment used to measure mechanical vibrations was a Svantek 948 m and two accelerometers (one for the measurement of full-body mechanical vibration, and the second for the exposure of the hand–arm segment). The acceleration results were evaluated, based on D.S. 594 [10], to determine if the vibrations to which the worker was exposed were classified as a risk, according to the Supreme Decree [9,11].

In [12], the authors presented a novel approach to vibration mitigation, which is based on a dynamic vibration absorbing structure (DVAS) for electric vehicles (EVs) that use switched reluctance motors (SRMs) at the wheels. The authors of [13] presented a systematic review studying professional drivers who are exposed to whole-body vibrations while driving, which contributes to an increased risk of developing physical problems, such as low back pain.

The study conducted in [14] compared the measurements obtained by the authors with established limits, aiming for future implementation of vibration control on the machine tool or in the receiving environment. The tool used for the measurement of mechanical vibrations in this study was the VI-400 Pro, which was analyzed by means of Quest Technologies, Product Design/Development, a 3M company, St. Paul, Minnesota, USA (QuestTM Professional II) and Microsoft Excel 2010.

The study in [15] consisted of the evaluation of mechanical vibrations during the driving of agricultural tractors and recreational vehicles (RVs). In both types of vehicles, measurements were made at different speeds. In agricultural tractors, they found that the maximum pressure of 0.80 MPa, recommended by ISO 2631-5:2004 [16], and the acceleration of 0.89 m/s${}^{2}$, recommended by ISO 2631-1: 1997 [1], were exceeded. In the RVs, they found the following data: the maximum pressure was less than 0.5 MPa, based on ISO 2631-5: 2004 [16]; and the maximum acceleration was less than 0.5 m/s${}^{2}$, according to ISO 2631-1: 2004 [17]. These data showed, according to the study, that, for an 8 h drive in recreational vehicles, there was no apparent risk; however, in agricultural tractors, there was a risk, as the recommended limits were exceeded.

The research project in [18] presented a study on vibration isolation, by means of the SEAT (seat effective amplitude transmissibility) factor, of four types of forklift crane seats with different vibration damping systems. In the study, to obtain the SEAT values, 30 min measurements were carried out for two conditions, with a 20 min travel time over a 6 km route. These data were compared with D.S. No. 594/1999 [10] and with DIRECTIVE 2002/44/EC [19] to determine the effect of the seats on mechanical vibration attenuation. The study showed that air suspension seats are the most effective ones, except for high crest factors, with an exposure below the permissible limits, according to D.S.No. 594/1999 [10]. The decree D.S.No. 594/1999 [10] is a regulation instituted by the Ministry of Health in Chile. We will incorporate references that adhere to this decree, which endorses the Statute on Fundamental Sanitary and Environmental Conditions in Workplaces. In Mexico, analogous decrees can be found in the following sources [20,21,22,23].

The studies reviewed in this section indicate a potential risk of developing health problems due to exposure to mechanical vibrations in certain types of vehicles. However, the risk of developing health problems can be reduced by using effective vibration isolation systems.

## 3. Methodology

Significant health consequences can arise for drivers after long working days driving trucks on Mexican roads, which have a deteriorated infrastructure. These conditions directly affect the driver’s body, as it is subjected to mechanical vibrations. The methodological proposal is based on the ISO 2631-1:1997 [1] standard, which is used to assess the vibration exposure of drivers over an average working day of 8 h.

**Figure 1:**
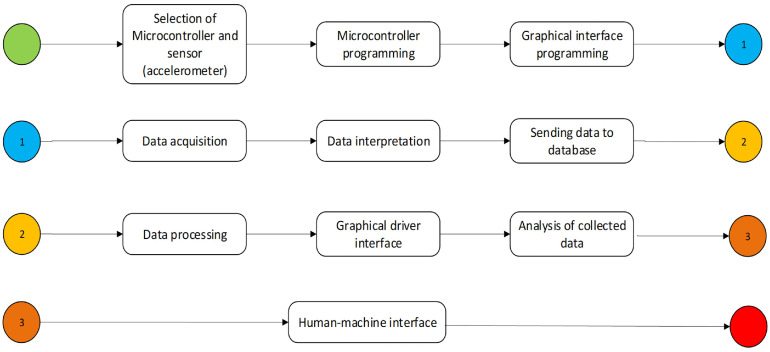
Block diagram of the design methodology of the research work.

Constant monitoring using IoT device technology enables early action to prevent long-term health damage. A methodology is proposed for the study and diagnosis of vibrations, which, through a human–machine interface, indicates to the driver the excess vibrations in their seat. Figure 1 shows the block diagram of the design methodology of the research work.

**Figure 2:**
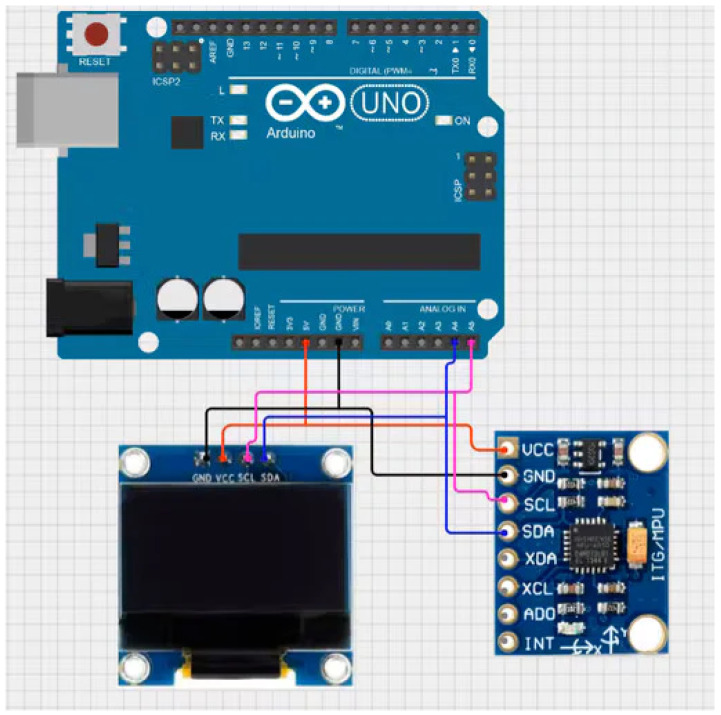
Logic diagram for data acquisition.

**Figure 3:**
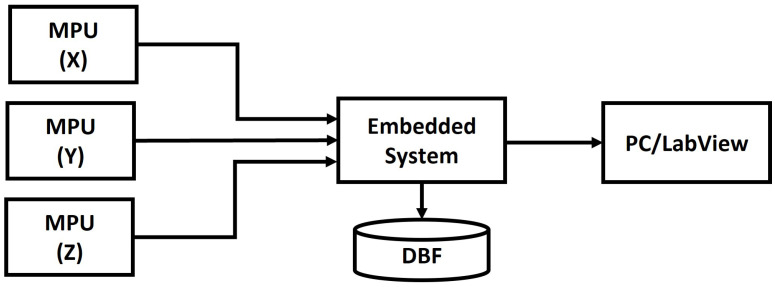
Diagram of the data acquisition process of the test prototype.

Figure 2 shows the logic diagram for the acquisition of vibration data from the driver’s seat using discrete IoT devices [24,25], and Figure 3 defines the diagram of the data acquisition process of the test prototype.

The initial data obtained from the sensor measurements are raw, with no scaling applied. The scaling technique implemented for the sensor data in its reading was derived from the resolution of the 16-bit ADC converter, with an accelerometer configuration of ±2 g. This results in the conversion to obtain the acceleration in m/s${}^{2}$. With this, the sensor, being initialized with the values of −2 g and +2 g, obtains accurate data for diagnostic information.

**Figure 4:**
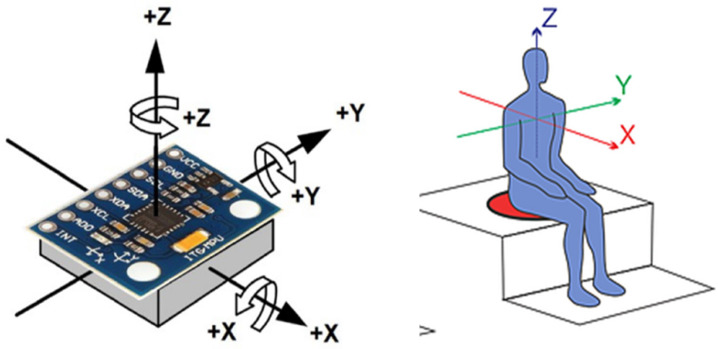
Arrangement of the axes according to ISO 2631 [1].

In the LabVIEW development environment, the data acquisition was programmed to send an identifier from the embedded system, plotting the three acceleration signals in their X-, Y-, and Z-axes across different graphs within a single cycle. Four Waveform Chart graphs were placed in the LabVIEW graphical interface to visualize the acquired data on the three axes as a function of time [26,27]. The layout of the axes, according to the positioning of the sensor in reference to ISO-2631 [1], is shown in Figure 4.

**Figure 5:**
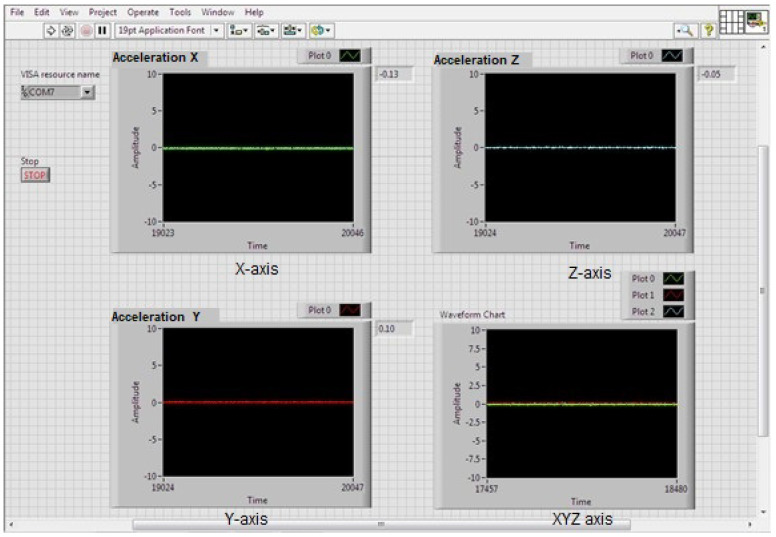
Signals from the three orthogonal axes, with acceleration readings at zero.

The accelerations obtained from the measurements on the three axes are shown in Figure 5.

**Figure 6:**
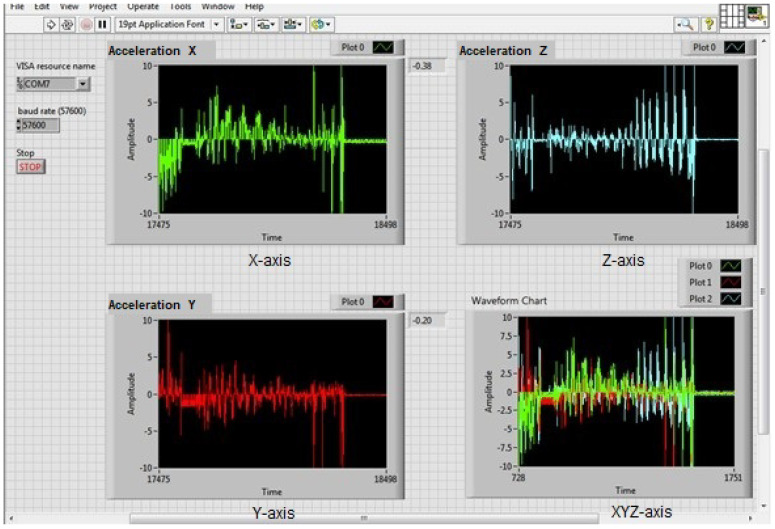
Accelerometer axis plots.

Figure 6 shows four plots, one for each axis of the accelerometer. The X-axis is shown in green, the Y-axis in red, and the Z-axis in blue. The final plot shows the three accelerations as a function of time. The vibration on the Z-axis is one of the mechanical vibrations that most affect the human body. This is because the Z-axis corresponds to the direction of gravity, so vibrations in this axis can cause the body to be shaken more forcefully.

**Figure 7:**
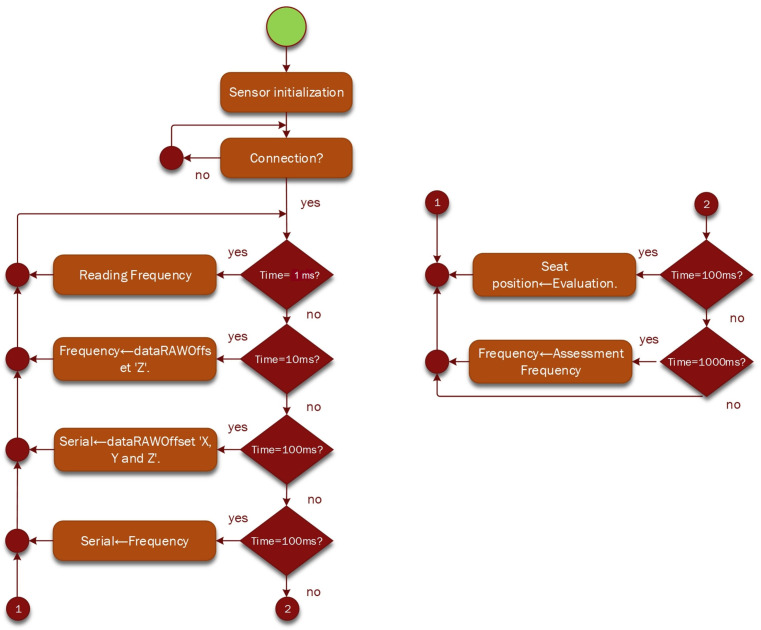
Data acquisition flowchart.

**Figure 8:**
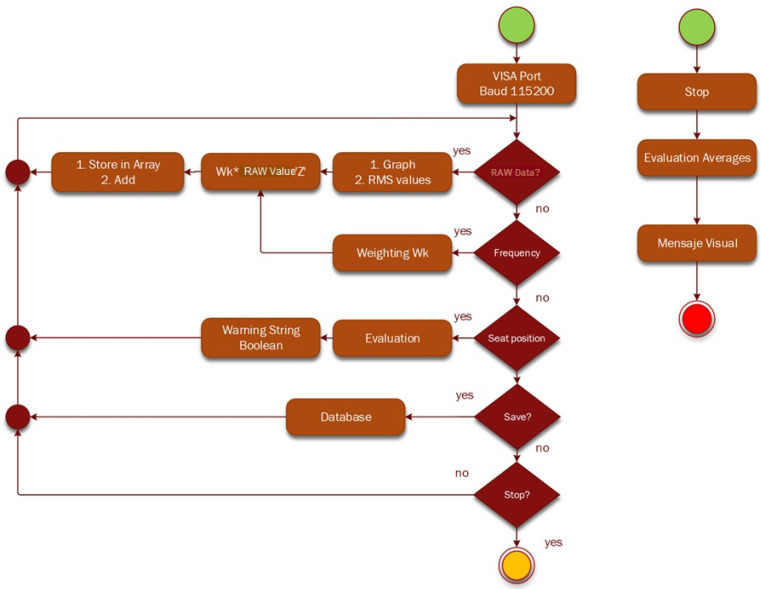
Flowchart for data processing in LabVIEW.

Figure 7 illustrates a flowchart of the data acquisition sequence, while Figure 8 presents a flowchart of the data processing in LabVIEW 2022 Q3 software, National Instrument, Austin, TX, USA.

According to the INSSBT (National Institute for Safety, Health, and Welfare at Work) and ISO-2631-1:1997 Amd 1:2010 [1], the criterion for the evaluation of mechanical vibrations is based on the calculation of the A(8) value through Equation (1) (A(8)-frequency-weighted effective acceleration referred to 8 h full body) in the orthogonal axes (X, Y, and Z). In this case, the Wk factor should only be taken into account for the orthogonal Z-axis. The obtained value must be compared with the value that triggers an action according to the set values.

The frequency-weighted acceleration on the Z-axis is obtained by Equation (1).

(1) $$a_{wk}=\sqrt{\sum_{i}^{}(w_{i}a_{i}}{)}^{2},$$

where:

- $a_{wk}$: frequency-weighted rms acceleration in the Z-axis,
- $a_{i}$: RMS acceleration value in Z,
- $w_{i}$: factor $w_{k}$.

For full-body transmitted vibration, the mentioned ISO standard states that frequencies between 0.5 and 80 Hz are the most damaging. However, the vibrations in this type of vehicle are in the range of 1–20 Hz, so a filter with a range of 1–30 Hz was implemented to observe the measured behavior. Another weighting factor established by the ISO-2631-1:1997 [1] standard is the $K_{i}$ factor, which varies according to the direction in which the vibration is produced. The $K_{i}$ factors for the X-, Y-, and Z-axes are $k_{x}=1.4$, $k_{y}=1.4$, and $k_{z}=1$, respectively.

Equation (2) is used for the evaluation of an 8 h exposure for the Z-axis:

(2) $$A_{z}\left(8\right)=k_{z}a_{wk}\sqrt{\frac{T}{8}},$$

where:

- $a_{z}$(8): frequency-weighted effective acceleration refers to an 8 h working day for the Z-axis (m/s)${}^{2}$,
- $k_{z}$: Z-axis factor,
- $a_{wk}$: frequency-weighted rms acceleration on the Z-axis,
- *T*: sample duration in hours.

**Figure 9:**
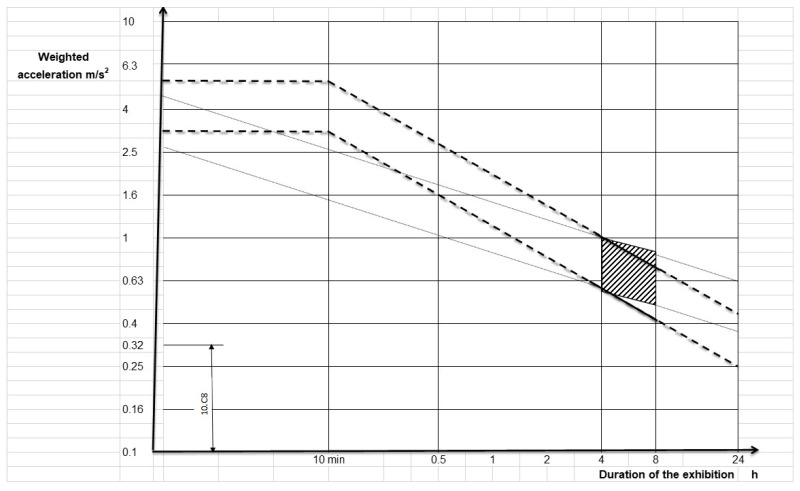
Health guidance for protection zones.

For health risk assessment, ISO 2631-1:1997 [1] provides the graph shown in Figure 9, which represents the permissible limits with respect to driving time exposure.

Vibrations must be measured in relation to a coordinate system, starting from a point from which the vibrations are considered to enter the human body. The vibration transmitted to the body must be measured on the surface, between the body and the surface. The main contact areas for seated persons are the seat support surface, the seat back, and the feet. For lumbar positions, the support surfaces are those under the pelvis, the back, and the head.

If it is not feasible to obtain precise alignment of the vibration transducers with the centric basic axes, the sensitive axes of the transducers may deviate from the reference axes by up to 15°. In addition to the weightings $w_{d}$ and $w_{k}$, multiplication factors, *K*, must also be applied, as shown below [1]:

- X−axis: $w_{d}$, K=1.4,
- Y−axis: $w_{d}$, K=1.4,
- Z−axis: $w_{k}$, K=1.

Equation (3) relates these parameters:

(3) $$A_{l}\left(8\right)=k_{l}\sqrt{\frac{1}{T_{0}}\sum_{i}^{}a_{wli}^{2}T_{i}}=k_{l}a_{wli}\sqrt{\frac{T_{i}}{T_{0}}}.$$

The calculation of A(8), for the axes, is expressed by the following equation (Equation (4)):

(4) $$A_{x}\left(8\right)=1.4a_{wx}\sqrt{\frac{T_{\exp}}{T_{0}}};A_{y}\left(8\right)=1.4a_{wy}\sqrt{\frac{T_{\exp}}{T_{0}}};A_{z}\left(8\right)=1a_{wz}\sqrt{\frac{T_{\exp}}{T_{0}}},$$

The evaluation of the vibration with respect to the acceleration, considering the highest frequencies present in each axis of the seat, is expressed by Equation (5):

(5) $$A\left(8\right)=\max[A_{x}\left(8\right),A_{y}\left(8\right),A_{z}\left(8\right)],$$

## 4. Results

The results obtained from the tests performed on the prototype developed and tested in a controlled environment that simulates mechanical vibration were recorded in a database for post-processing. The data are shown in Table 1. It is important to observe that this is just a representative subset of the total data collected for this study, comprising approximately 5% of the total dataset. This was done to provide a snapshot of the data without overwhelming the reader with the sheer volume of measurements collected. However, additional tables that encompass the complete set of measurements are available as additional material for this paper: Measurement Database (https://drive.google.com/drive/folders/1Dr7M9_Udb4SVWBwtUVPsOMG8XSo_WxDO?usp=sharing (accessed on 10 November 2023).

The two main factors affecting driver health, as mentioned above, are acceleration and frequency. Therefore, each of these indicators must be analyzed separately. For the purposes of studying an 8-hour working day, they will be analyzed as an average, as per ISO 2631-1:1997-1997/Amd 1:2010 [1].

### 4.1. Acceleration

The data obtained from the accelerometer are initially the raw data directly from the ADC converter of the accelerometer. These data are then processed by the digital motion processor (DMP) and acquired via serial communication.

#### Analysis of Acceleration without Mechanical Vibration

**Figure 10:**
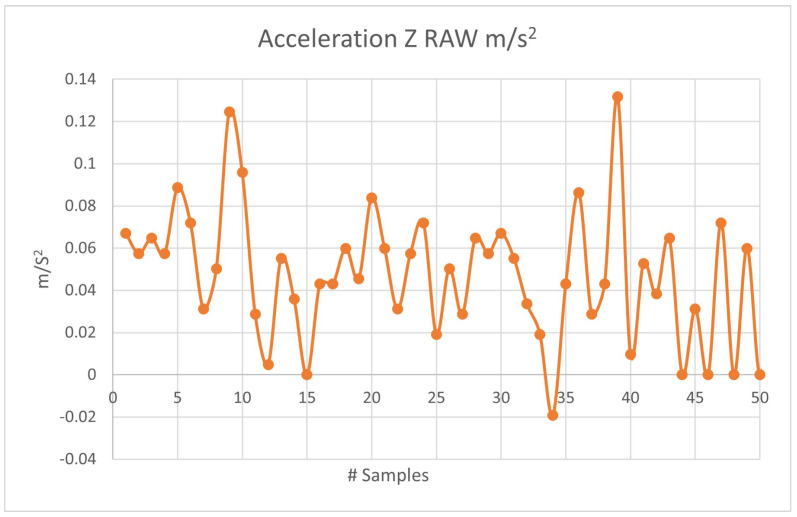
RAW acceleration data in m/s${}^{2}$ Z-axis: 50 samples.

Figure 10 shows part of the data acquisition in the Z-axis at a time of 5 s. The data were processed using the LabVIEW 2022 Q3 software, National Instrument, Austin, TX, USA.

**Figure 11:**
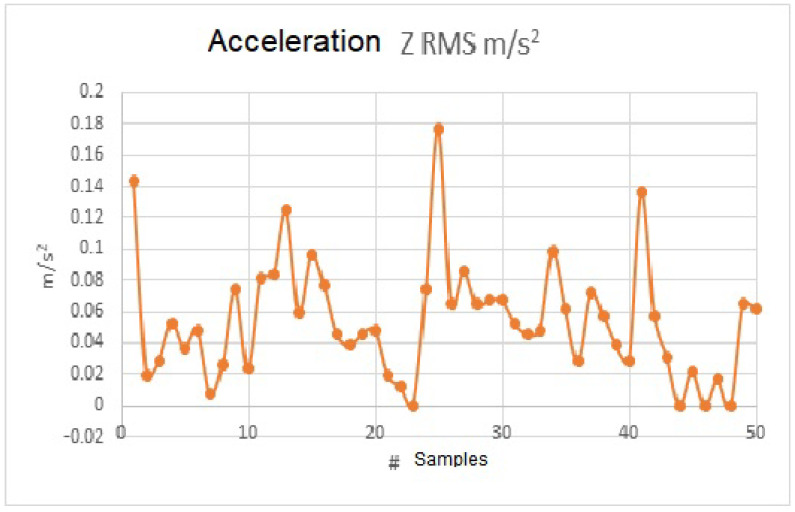
RMS acceleration data in m/s${}^{2}$ Z-axis: 50 samples.

This sample was taken from the sensor while it was completely horizontal and without any mechanical vibration that could alter the acceleration measurement. Figure 10 shows that there is an acceleration ranging from −0.02 m/s${}^{2}$ to 0.13 m/s${}^{2}$. A filter was implemented for the frequency to reduce noise in the measurement and eliminate erroneous data on the frequency at which it is vibrating. The filter implemented is a type that cancels out noise data within ±0.2 m/s${}^{2}$, obtained from the accelerometer. This acceleration data were processed in LabVIEW to obtain the RMS values. Figure 11 shows the new data obtained as a function of time.

Figure 11 shows the RMS acceleration data from Figure 10 with RMS values. As can be seen, the values received from the raw sensor in m/s${}^{2}$ are being processed by the VI basic RMS, obtaining an output of RMS values. In the following process, these values can be weighted according to their frequency.

**Figure 12:**
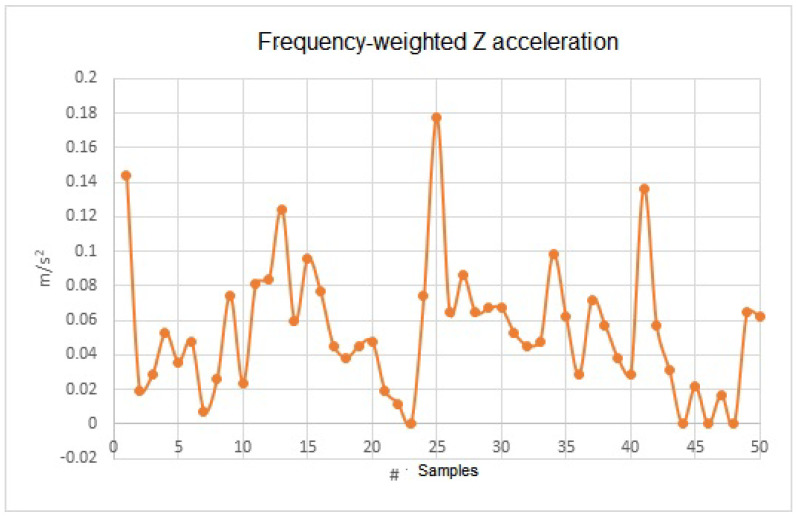
Frequency-weighted acceleration for the data.

In Figure 12, we can observe the similarity of the data obtained, even when they are frequency-weighted. This is because the frequency filter is acting. Since the measurement was taken with the sensor completely horizontal and without inducing any mechanical vibration, the RMS data are simply multiplied by a factor of 1, which gives the same result.

### 4.2. Analysis of Acceleration and Frequency Data

With the correct functioning of the filter (frequency-weighted acceleration), the sensor was placed in a prototype machine, in which a mechanical vibration, measured by the sensor, was induced. The measurement results were recorded in a database of the three orthogonal axes.

#### 4.2.1. X-axis Data

**Figure 13:**
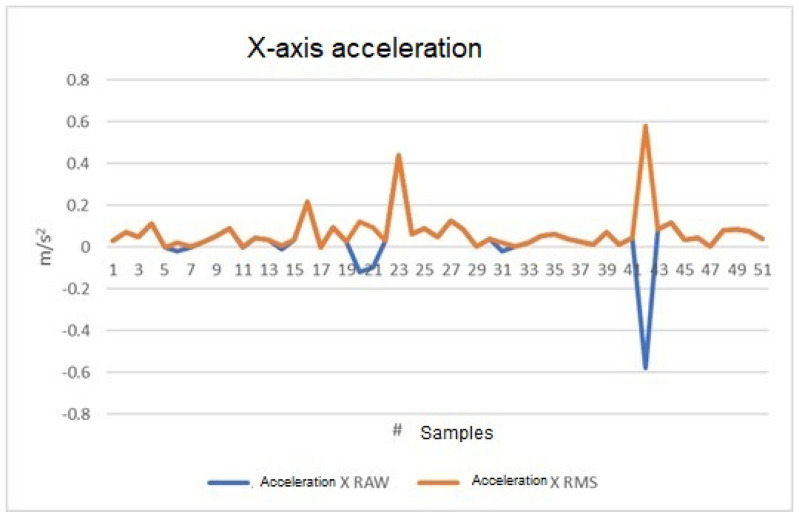
X-axis acceleration, RAW, and RMS values in m/s${}^{2}$.

**Figure 14:**
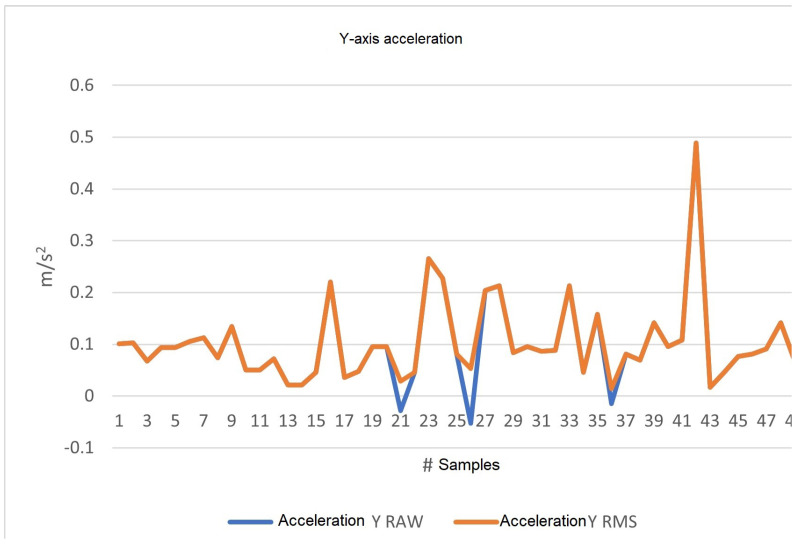
Y-axis acceleration, RAW, and RMS values in m/s${}^{2}$.

**Figure 15:**
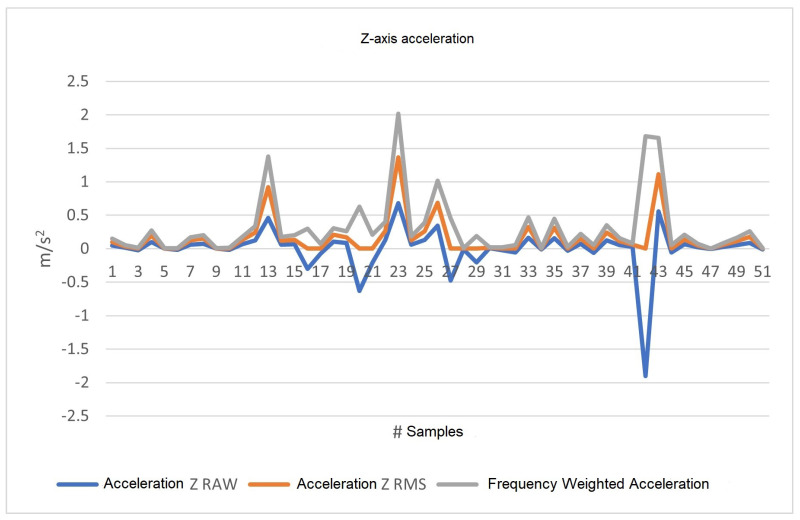
Z-axis acceleration, RAW, RMS, and frequency-weighted values in m/s${}^{2}$.

Figure 13 depicts data captured on the X-axis. The blue line signifies data measured in m/s${}^{2}$, gathered by the accelerometer and transmitted to the LabVIEW development software via a serial protocol. These data are processed by the software, generating the orange graph that displays the RMS values. Notably, this processing analyzes the data without considering frequency weighting. This graph compares the behavior of the provided information, and the same reasoning applies to the Y- and Z-axes in Figure 14 and Figure 15.

#### 4.2.2. Y-axis Data

As shown in Figure 14, with the same number of samples and sampling time as the previous X-axis analysis, the raw data in m/s${}^{2}$ are shown in blue. When processed by the software, these data are plotted in orange with RMS values.

#### 4.2.3. Z-axis Data

As can be seen in Figure 15, the three lines represent the acceleration according to the type of processing being carried out. The blue line represents the acceleration in m/s${}^{2}$ with the direct values from the accelerometer. The orange line represents RMS data, and, finally, the frequency-weighted acceleration data are represented in the gray color. These data are obtained according to the arrangement described above, obtaining the frequency ratio and the $w_{k}$ factor.

In Figure 13, Figure 14 and Figure 15, we plot acceleration on the three axes. The blue line (RAW values) shows an increase when the vehicle accelerates and a decrease when it decelerates, explaining the negative values. Conversely, the orange line (RMS values) always displays positive values, as RMS values are calculated by taking the square root of the mean of the squares of the acceleration values, and are always positive by definition. Therefore, when the vehicle decelerates, the plot of both values (RAW and RMS) will show discrepancies, indicating that the RMS plot does not consider whether the vehicle is accelerating or not, only the energy or intensity of the acceleration. This also explains why the orange value at those points is not equivalent to simply removing the sign of the signal. Figure 13 and Figure 15 show more discrepancies, indicating frequent acceleration and deceleration in the X- and Z-axes, which is expected as they reflect the car’s forward movement or stopping, and vibrations, respectively. Figure 14 appears less discrepant, aligning with the observation that there are virtually no sudden changes in acceleration from the driver’s left or right side (the Y-axis).

**Figure 16:**
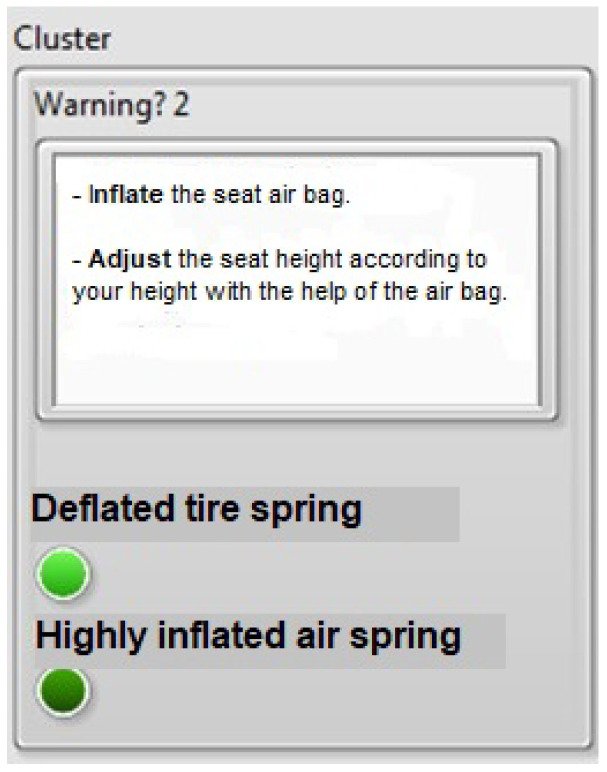
Man–machine interface with the message: deflated air spring.

**Figure 17:**
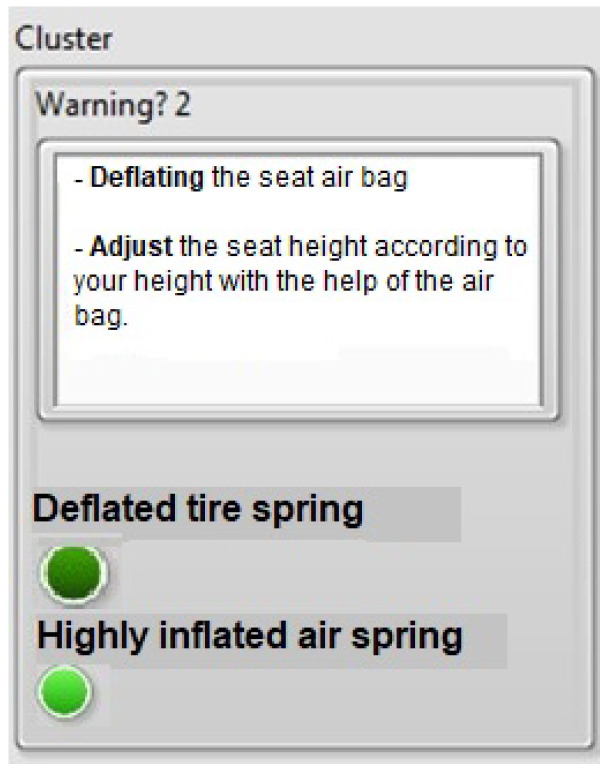
Man–machine interface with the message: over-inflated air spring.

The results obtained by this prototype in a controlled environment, when applied to the field, will allow us to verify the stability of the components under various conditions that occur in a tractor-trailer cabin and to confirm the robustness of the system. The graphical user interface was developed in a driver-friendly manner, regardless of the database, to provide the driver with warnings and possible solutions to perceived mechanical vibrations. Additionally, a graphical user interface was developed to address the inconvenience of impacts caused by seating position, using easy-to-understand language for the driver. Both graphical interfaces can be integrated with current technology, as most vehicles have built-in displays. This is illustrated in Figure 16 and Figure 17, which alert the driver if the seat is in either of two states.

Case 1: Deflated pneumatic spring.

The driver will receive two indications to prevent the seat from entering this state:

1. INFLATE the seat airbag. This first message, in conjunction with the LED indicator, informs the driver that the seat is in its lowest position. As a result, there is no damping, and mechanical vibrations are transferred without being attenuated by the seat suspension;
2. Adjust the seat height according to the driver’s height using the airbag. The driver is then advised of the action to take to ensure proper damping.

Case 2: Over-inflated tire spring

Similarly, the driver will receive two indications to prevent the seat from entering this state:

1. DEFLATE the seat airbag. In addition to the LED indicator, the driver is warned that the seat is in its highest position, resulting in the same consequences as in case one;
2. Adjust the seat height according to the driver’s height using the airbag. The driver then receives a recommendation for the action to take to ensure proper cushioning.

## 5. Discussion

ISO 2631-1:1997 [1] is a widely recognized standard used in the diagnosis and study of mechanical vibrations in cargo vehicles. This standard establishes criteria for assessing human exposure to vibrations transmitted through vehicle seats, with the aim of preventing possible adverse health effects on drivers. It provides a standardized and objective approach to vibration assessment, enabling appropriate corrective and preventive measures to be taken to minimize negative effects on drivers. By using this standard, analysis can be performed to assess compliance with established criteria. Additionally, the standard provides guidelines for designing and manufacturing vehicle seats, with the aim of reducing vibration transmission to the human body. These criteria help determine whether vibrations are within acceptable limits or require corrective actions to reduce health risks. In this paper, we consider aspects such as sensor response, measurement of collected information, surface types, real experimental environment, effects of noise on sensors, dynamics of real environments, and reliability and efficiency of approaches for real-time operation. We also present a summary of the strengths and challenges of the considered approaches. Strengths define the main contributions of each approach to avoid response errors. Challenges remain for successfully solving problems or assessing effectiveness when applied under conditions not considered in this research. An important observation is that we have determined that most vehicles exhibit vibrations of minor or major scale.

This work concentrates on diagnosing and studying the mechanical vibrations produced in a cargo vehicle while adhering to ISO 2631 [1]. An additional step is to consider security measures for data protection and end-user safety, such as the drivers of freight vehicles [13,28,29,30,31,32,33,34,35,36]. These works concern the investigation, analysis, and forecasting of the impact of vibration on cargo vehicle drivers. Safety is also examined in other fields, such as public health and industrial safety. Safety standards in Mexico are proposed, studied, and implemented by various government entities, including Secretariats, Commissions and Standardization Centers, in addition to these standards.

These regulations, specifications, standards, and laws, which have been issued by the Mexican State, dictate the legal framework in the United Mexican States that all vehicle owners, manufacturers, and end-users must abide by through the Presidency of the Republic [20,21,22,23].

The effectiveness of our system is demonstrated through the continuous diagnosis and study of mechanical vibrations present during driving. This effectiveness is not solely dependent on the driving time or road conditions. It also extends to scenarios where the occurrence of mechanical vibrations increases, both with and without preventive measures. This allows our system to maintain a high level of effectiveness, whether the driving time is long or short, whether the road conditions are good or poor, and whether preventive measures are in place or not. The supplementary material, which includes additional measurements, allows for the examination of various driving times and conditions.

## 6. Conclusions

The detection, measurement, and prevention of mechanical vibrations generated by various factors in the seat of a cargo vehicle can be achieved in several ways. One proposed solution is through constant monitoring of mechanical vibrations. Upon reviewing current regulations on whole-body mechanical vibrations, it was found that the ISO 2631-1:1997 [1] standard specifies the methods for measurement, weightings, and equations necessary to determine when mechanical vibrations exceed a parameter dependent on frequency and acceleration. By considering the points mentioned in the ISO 2631-1:1997 [1] standard, a prototype was developed to measure variables using an MPU-6050 accelerometer and appropriate manipulation of raw acceleration data acquired through the I2C communication protocol. An embedded system was used to manipulate acceleration data to obtain a vibration frequency in a range of 1–30 Hz on the Z-axis. The focus was on the Z-axis, as suggested by the ISO standard, due to its positioning in the driver’s seat and its likelihood of aggravating mechanical vibrations due to impacts. The data obtained from the sensor were successfully transmitted via serial protocol to LabVIEW software [26,27]. With this software, diagnosis of Z-axis data in frequency weighting was developed; for the other two axes, only RMS values were obtained. The diagnosis of the information led to the presentation of results to a man–machine interface, as an indication to the driver of actions to be taken.

In the future, we could expect the diagnosis and study of mechanical vibrations in cargo vehicles to evolve as follows:

1. Advanced monitoring technology: as sensor and monitoring technology continues to advance, it is possible that more sophisticated systems will be developed to collect and analyze real-time vibration data on cargo vehicles. This would allow early detection of problems and more accurate assessment of vibration exposure;
2. Data integration: integration of vibration data with other in-vehicle diagnostic systems (such as onboard diagnostic systems, telemetry, etc.) could enable a more complete and rapid assessment of the mechanical health of cargo vehicles;
3. Optimizing comfort and safety: As concerns for the well-being of drivers and passengers increase, systems may be developed that use vibration information to optimize comfort and safety in cargo vehicles, reducing driver fatigue and improving the user experience;
4. Adaptation to changing regulations: regulations regarding vibration exposure in work environments and in vehicles may change over time. Standards, including ISO 2631-1:1997 [1], may be updated to reflect new knowledge and safety standards;
5. Focus on prevention: as the industry becomes more proactive in preventive maintenance, vibration studies could be used to identify potential problems before they become serious failures, which could increase the lifetime of cargo vehicles and reduce maintenance costs.

An interesting avenue for future work could be to explore the interaction between vehicle acceleration and driver response in real-world scenarios, including those with rough road surfaces and emergency stop or start conditions. This would involve investigating how both acceleration and vibration affect the driver, providing a more comprehensive understanding of the driving experience. Obtaining real-world data under these conditions would allow us to confirm the trends observed in the laboratory and assess the validity of our prototype. The data were generated synthetically in the laboratory through a prototype measurement for the diagnosis and study presented. However, this would require a new approach and methodology, distinct from the focus of our current paper. We look forward to delving into these aspects in our future research endeavors.

## Figures and Tables

**Table 1:** Records obtained and stored.

# Sample	Day and Time of the Sample	Acceleration Raw (m/s^2^)			Acceleration RMS (m/s^2^)			Frequency	Wk	Frequency Weighted Acceleration
		**X**	**Y**	**Z**	**X**	**Y**	**Z**	**Hz**		**m/s^2^**
1	25/05/2018 11:49:49 p.m.	0.01437	0.107776	0.579595	0.01437	0.107776	0.579595	30	0.429	0.248646
2	25/05/2018 11:49:49 p.m.	−0.011975	0.02395	0.00479	0.011975	0.02395	0.000479	30	0.429	0.002055
3	25/05/2018 11:49:49 p.m.	0.05748	0.01916	−0.546064	0.05748	0.01916	0.545064	30	0.429	0.234262
4	25/05/2018 11:49:49 p.m.	0.021555	0.088616	−0.076641	0.021555	0.088616	0.076641	30	0.429	0.032879
5	25/05/2018 11:49:49 p.m.	−0.095801	0.062271	0.65863	0.095801	0.062271	0.65863	21	0.611	0.402423
6	25/05/2018 11:49:50 p.m.	−0.02874	0.062271	−0.134121	0.02874	0.062271	0.134121	21	0.611	0.081948
7	25/05/2018 11:49:50 p.m.	0.040715	0.05748	−0.713716	0.040715	0.05748	0.713716	21	0.611	0.43608
8	25/05/2018 11:49:50 p.m.	−0.03335	0.074246	0.086221	0.03353	0.074246	0.086221	21	0.611	0.052681
9	25/05/2018 11:49:50 p.m.	−0.074246	0.059875	0.500559	0.074246	0.059875	0.500559	21	0.611	0.305842
10	25/05/2018 11:49:50 p.m.	−0.02395	0.086221	−0.160466	0.02395	0.086221	0.160466	21	0.611	0.098045
11	25/05/2018 11:49:50 p.m.	−0.007185	0.05269	−0.522114	0.007185	0.05269	0.522114	21	0.611	0.319012
12	25/05/2018 11:49:50 p.m.	−0.031135	0.035925	0.126936	0.031135	0.035925	0.126936	21	0.611	0.077558
13	25/05/2018 11:49:50 p.m.	−0.02395	0.143701	0.546064	0.02395	0.143701	0.546064	21	0.611	0.333645
14	25/05/2018 11:49:50 p.m.	0.002395	0.105381	−0.241897	0.002395	0.105381	0.241897	21	0.611	0.147799
15	25/05/2018 11:49:50 p.m.	0.00958	0.124541	0.531694	0.00958	0.124541	0.531694	0	1	0.531694
16	25/05/2018 11:49:51 p.m.	−0.088616	0.141306	0.134121	0.088616	0.141306	0.134121	0	1	0.134121
17	25/05/2018 11:49:51 p.m.	−0.107776	0.098196	0.411943	0.107776	0.098156	0.411943	0	1	0.411943
18	25/05/2018 11:49:51 p.m.	−0.016765	0.110171	−0.277822	0.016765	0.110171	0.277822	0	1	0.277822

## Data Availability

The data base of the laboratory measurements are available at: (https://drive.google.com/drive/folders/1Dr7M9_Udb4SVWBwtUVPsOMG8XSo_WxDO?usp=sharing (accessed on 10 November 2023).

## Abbreviations

The following abbreviations are used in this manuscript:

ISO	International Organization for Standardization
GUI	Graphical user interface
SEAT	Effective seat width transmittance
ACHS	Chilean Safety Association
SDA	Speaker drive amplifier
SCL	System clock
ADC	Analogue-to-digital converter
INSSBT	National Institute for Safety, Health and Welfare at Work
DMP	Digital motion processor
RAW	RAW sensor data
RMS	Root mean square
RVs	Recreational vehicles
MPa	Megapascal
LabVIEW	Laboratory Virtual Instrument Engineering Workbench
LED	Light-emitting diode
